# Personal independence payments among people who access mental health services: results from a novel data linkage

**DOI:** 10.1192/bjo.2024.68

**Published:** 2024-09-24

**Authors:** Sharon A. M. Stevelink, Ioannis Bakolis, Sarah Dorrington, Johnny Downs, Ray Leal, Ira Madan, Ava Phillips, Ben Geiger, Matthew Hotopf, Nicola T. Fear

**Affiliations:** Department of Psychological Medicine, Institute of Psychiatry, Psychology and Neuroscience, King's College London, UK; and King's Centre for Military Health Research, Department of Psychological Medicine, Institute of Psychiatry, Psychology and Neuroscience, King's College London, UK; NIHR Maudsley Biomedical Research Centre, South London and Maudsley NHS Foundation Trust, London, UK; Department of Biostatistics and Health Informatics, Institute of Psychiatry, Psychology and Neuroscience, King's College London, UK; and Centre for Implementation Science, Health Service and Population Research Department, Institute of Psychiatry, Psychology and Neuroscience, King's College London, UK; Department of Psychological Medicine, Institute of Psychiatry, Psychology and Neuroscience, King's College London, UK; and NIHR Maudsley Biomedical Research Centre, South London and Maudsley NHS Foundation Trust, London, UK; Department of Occupational Health, Guy's and St Thomas’ Hospitals NHS Trust, London, UK; Department of Psychological Medicine, Institute of Psychiatry, Psychology and Neuroscience, King's College London, UK; Centre for Society and Mental Health, Institute of Psychiatry, Psychology and Neuroscience, King's College London, UK; King's Centre for Military Health Research, Department of Psychological Medicine, Institute of Psychiatry, Psychology and Neuroscience, King's College London, UK; and Academic Department of Military Mental Health, Department of Psychological Medicine, Institute of Psychiatry, Psychology and Neuroscience, King's College London, UK

**Keywords:** Big data, mental health services, epidemiology, register-based epidemiology, register-based study

## Abstract

**Background:**

Personal independence payment (PIP) is a benefit that covers additional daily living costs people may incur from a long-term health condition or disability. Little is known about PIP receipt and associated factors among people who access mental health services, and trends over time. Individual-level data linking healthcare records with administrative records on benefits receipt have been non-existent in the UK.

**Aims:**

To explore how PIP receipt varies over time, including PIP type, and its association with sociodemographic and diagnostic patient characteristics among people who access mental health services.

**Method:**

A data-set was established by linking electronic mental health records from the South London and Maudsley NHS Foundation Trust with administrative records from the Department for Work and Pensions.

**Results:**

Of 143 714 working-age patients, 37 120 (25.8%) had received PIP between 2013 and 2019, with PIP receipt steadily increasing over time. Two in three patients (63.2%) had received both the daily living and mobility component. PIP receipt increased with age. Those in more deprived areas were more likely to receive PIP. The likelihood of PIP receipt varied by ethnicity. Patients diagnosed with a severe mental illness had 1.48 odds (95% CI 1.42–1.53) of having received PIP, compared with those with a different psychiatric diagnosis.

**Conclusions:**

One in four people who accessed mental health services had received PIP, with higher levels seen among those most likely in need, as indicated by a severe mental illness diagnosis. Future research using this data-set could explore the average duration of PIP receipt in people who access mental health services, and re-assessment patterns by psychiatric diagnosis.

About one in in five working-age people in the UK self-report a long-term physical or mental health condition, or a disability, that may interfere with their daily lives.^1^ For many people, this seriously affects their mobility and ability to undertake everyday activities. The UK Government provides financial support in the form of a benefit, personal independence payment (PIP), which is specifically aimed at covering some of the additional costs that people in England may incur because of their disability or long-term health condition.^2^ PIP was introduced in 2013, as part of the Welfare Reform Act 2012, and in 2023, 3 million people received PIP.^3^ For the working-age population, PIP has largely replaced a relatively similar pre-existing benefit, disability living allowance (DLA), but DLA is still received by an additional 1.3 million people, mostly children and older people.^4^ In 2021–2022, the UK Government spent £15 billion on PIP.^5^ PIP consists of two components, a daily living component if someone needs help with daily tasks, and a mobility component if someone needs help with getting around. PIP is a non-means-tested benefit, meaning that it does not matter what an individual's income or wealth is: people can claim PIP whether or not they are working. Those who apply for PIP will have to undergo a PIP assessment. This assessment is focused on determining the functional impact of the disability or long-term health condition, and its outcome will inform whether someone is eligible for PIP and the amount they qualify for.^6^ Each component has two payment rates, standard and enhanced. Once awarded, the Department for Work and Pensions (DWP) can stipulate a re-assessment of a person's entitlement to PIP at any time, even if the initial PIP award was only granted for a fixed duration of time. The DWP is responsible for the implementation and administration of welfare policies, including benefits payments.

## PIP and mental health

Previous qualitative research among people with mental health problems has shown that the PIP assessment may not be tailored to the fluctuating nature of mental disorders and that the process of applying is experienced as stressful.^7,8^ This is particularly pertinent as the UK Government has recently announced that it will expand the use of the PIP assessment across a variety of benefits, including replacing the work capability assessment (WCA) that people currently undergo as part of the claims process for out-of-work benefits.^9^ This means that in the future, one single assessment may be used to inform people's eligibility across a whole range of benefits. By doing this, the government aims to encourage more people with longstanding health conditions and disabilities into work, as certain out-of-work benefits (e.g. Universal Credit) are no longer directly linked to a person's capability to work. It is important to note that these changes are planned to be rolled out only from 2026/2027 onward. Before the proposed reforms, people who were assessed as being unable or having limited capability to work were eligible to receive an additional ‘limited capability for work-related activity payment’ (LCWRA) in addition to the standard Universal Credit allowance. The LCWRA payment will be replaced by a Universal Credit health element, but people are only eligible to receive this if they qualify for both PIP and the standard Universal Credit payment. Consequently, people who receive the LCWRA as part of their Universal Credit but who do not receive PIP, may no longer qualify for the Universal Credit health element if their difficulties are not deemed to incur additional daily living and mobility costs. On the other hand, those who currently receive PIP, but not the Universal Credit health element, may benefit as they could be deemed eligible to receive the Universal Credit health element as well.^9,10^

National data show that the most frequently reported disabling conditions underpinning an individual's PIP claim are psychiatric disorders (37%).^3^ However, to date, very little research has looked at the sociodemographic and diagnostic profile of people who access mental health services and trends over time since the transition from DLA to PIP. This is because there has been little interchange of individual-level health and administrative data, as in general, these types of data are kept separately in the UK. To address this, we have established the first data linkage between electronic mental health records from one of the largest European mental health service providers with administrative records concerning benefits receipt from the DWP. The current study aimed to explore (a) how PIP receipt, including type of PIP (e.g. daily living, mobility or both), varied over time among people who use mental health services; and (b) the associations between sociodemographic and diagnostic patient characteristics and PIP receipt.

## Method

### Data source and linkage

A linked data source was established using an *ad hoc* deterministic linkage method, combining electronic mental health records from the South London and Maudsley NHS Foundation Trust (SLaM) with administrative records from the DWP (linkage rate 92.3%).^11^ SLaM electronic mental health record data were extracted via the Clinical Record Interactive Search (CRIS) system.^12,13^ Individual-level patient data, derived from structured fields, included sociodemographic characteristics (month and year of birth, ethnicity, deprivation, area of residence), referral data (time in SLaM) and diagnostic data (recorded primary psychiatric diagnosis, severe mental illness (SMI) diagnosis). Administrative data from DWP included individual level data on gender and mortality, as well as the individual's start and end dates on a range of benefits, in particular PIP.

### Study population

The sample included adults of working age who were referred to SLaM mental health services between 2007 and 2019, and who had their electronic mental health records successfully linked with administrative records from DWP. The majority of the sample accessed SLaM secondary mental health services, but some patients were also referred to the national service provision SLaM provides (e.g. tertiary mental health services). In practice, this meant that adults who had a national insurance number and were successfully linked, formed part of the linked data-set, including those who had never applied for, or never received, any benefits. Working age was defined as individuals who were aged 18 years at the start of the PIP data window (January 2013) and below 67 years at the end of the PIP data window (December 2019). Patients who died before the introduction of PIP in 2013 were excluded. SLaM provides local mental health services to residents living in four boroughs in South London, UK, as well as national (specialist) services to those who are referred into SLaM from across the country. The SLaM catchment area is a high-density urban area with an ethnically diverse population, with pockets of both affluence and poverty.

### Statistical analysis

The study had a cross-sectional design and the planned statistical analysis approach was pre-registered (https://osf.io/3pgty). No deviations from the analysis protocol were noted. All statistical analyses were conducted in Stata for Windows version 17.0. A sociodemographic and diagnostic profile of the included patient sample was provided with descriptive statistics. Subsequently, the number of patients receiving PIP, in general and by type of PIP (daily living only, mobility only or both), on a calendar year basis between 2013 and 2019 was determined and depicted graphically. Univariable and multivariable logistic regression analyses were conducted with PIP receipt (irrespective of the type) as the outcome of interest, thereby exploring associations with sociodemographic and diagnostic characteristics. An *a priori* decision was made to adjust for age, gender, ethnicity, deprivation and recorded primary psychiatric diagnosis, informed by a scoping review of the literature and expert opinion.^11,14^ A planned sensitivity analysis was conducted exploring whether the results of the logistic regression analyses for PIP receipt (irrespective of type of PIP) differed substantially if these were restricted to patients who lived in the SLaM catchment area. This was done in anticipation of a different sociodemographic and psychiatric diagnosis typology of patients who were referred to SLaM to access national specialist services. As planned, we created an ethnicity category combining ‘not stated’, ‘unknown’ and ‘missing’, to preserve our sample in the multivariable analyses. Multinomial logistic regression analyses were conducted to explore associations between sociodemographic and diagnostic characteristics with the type of PIP (daily living only, mobility only or both) as the outcome of interest. A proportion of patients had a ‘nil’ outcome in their PIP status. This means that they were not awarded the type of PIP they applied for or that the PIP claim review was in process. We did not consider this information in our analysis.

### Ethical approval

The authors assert that all procedures contributing to this work comply with the ethical standards of the relevant national and institutional committees on human experimentation and with the Helsinki Declaration of 1975, as revised in 2008. All procedures involving patients were approved by the South Central – Oxford C Research Ethics Committee (approval references 17/SC/0581, 22/SC/0400 and 23/SC/0257). Section 251 approval was provided by the NHS Health Research Authority Confidential Advisory Group (approval reference 17CAG0055), as it was not deemed practical or feasible to obtain informed consent from all patients.

## Results

**Fig. 1:**
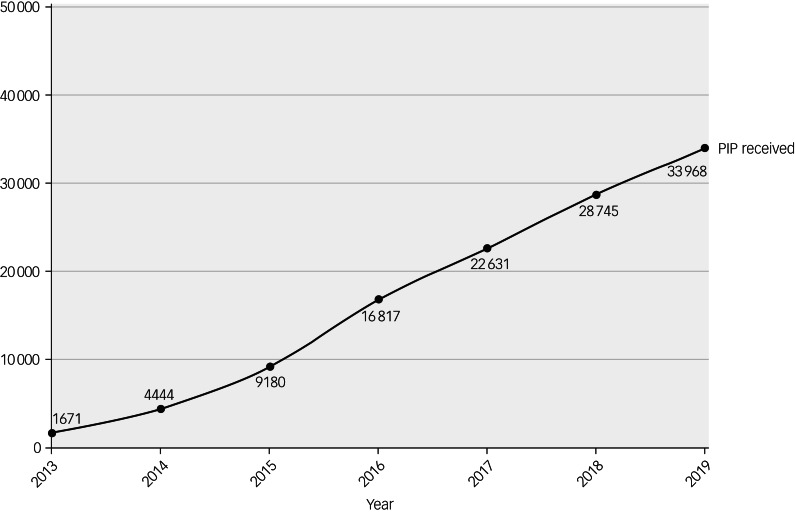
Number of patients who received PIP (irrespective of type of PIP) by calendar year (*N* = 143 714, of whom *n* = 37 120 had received PIP), data covering 2013–2019. PIP, personal independence payment.

**Fig. 2:**
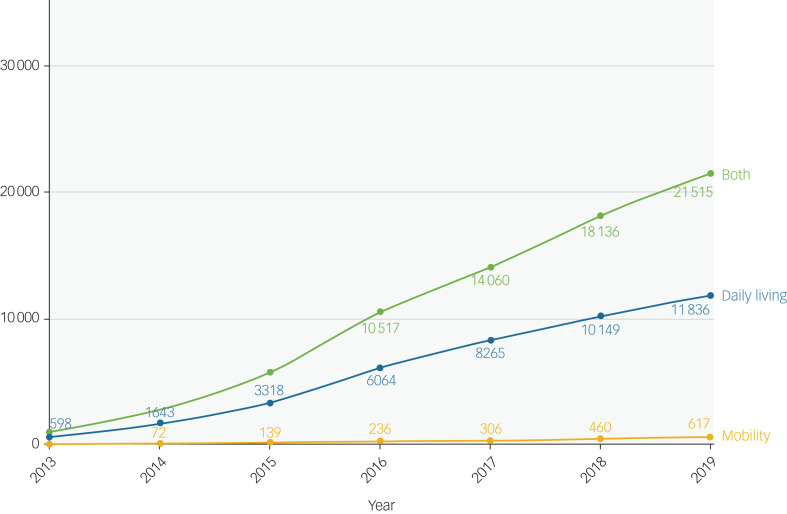
Number of patients who received PIP (daily living only, mobility only or both) by calendar year (*N* = 143 714, of whom *n* = 37 120 had received PIP), data covering 2013–2019. PIP, personal independence payment.

The average age of the 143 714 included patients was 36.7 years (s.d. 11.1), and over half of the patients were from a White ethnic background (Table 1). Levels of deprivation were substantial, as 66.6% of the patients were in the two most deprived quintiles. Two-thirds of patients had resided in the SLaM catchment area. A total of 25.8% of patients (*n* = 37 120) had received PIP between 2013 and 2019. Most patients had received both the daily living and mobility component of PIP (*n* = 23478, 63.2%). Just 2.0% of patients had only received a mobility award. A sustained increase in PIP receipt was noted over time, with 1671 patients (1.2%) in receipt of PIP in 2013, compared with 33 968 (23.6%) in 2019 (Figs 1 and 2).

**Table 1:** Profile of patients included in the study (*N* = 143 714)

Characteristic	Total *n*	%
Overall	143 714	
Gender		
Female	69 477	48.3
Male	74 237	51.7
Age (years)^a^		
Mean (s.d.)	36.7 (11.1)	
18–24	24 681	17.2
25–34	41 376	28.8
35–44	36 233	25.2
45–54	32 331	22.5
55–66	9093	6.3
Ethnicity		
White	65 253	54.6
Black/African/Caribbean/Black British	20 514	17.2
Asian/Asian British	3123	2.6
Mixed/multiple racial and ethnic groups	3041	2.5
Other racial and ethnic minority groups	192	0.2
Not stated	27 368	22.9
Deprivation (IMD quintile)^b^		
First (most deprived)	43 196	31.5
Second	48 019	35.1
Third	25 565	18.7
Fourth	12 379	9.0
Fifth (least deprived)	7832	5.7
Ever resident within local catchment area^c^		
No	43 008	31.0
Yes	95 660	69.0
Death		
No	133 914	93.2
Yes	9800	6.8
Primary psychiatric diagnosis recorded^d^		
No	46 691	32.5
Yes	97 023	67.5
Total number of days active in SLaM^e^		
Median days (IQR)	368	(IQR 63–1341.5)
Received PIP		
No	106 594	74.2
Yes	37 120	25.8
If received PIP, which type^f^		
Only daily living	12 912	34.8
Only mobility	730	2.0
Both	23 478	63.2

IMD, Index of Multiple Deprivation; SLaM, South London and Maudsley NHS Foundation Trust; IQR, interquartile range; PIP, personal independence payment.

a. Calculated at the PIP window start date (January 2013).

b. IMD scores published in 2015, patient postcode used closest before or after the PIP window start date (January 2013).

c. Defined as recorded at least one patient postcode within the SLaM catchment area within study window.

d. Earliest available within study window (January 2007 to December 2019), based on ICD-10 ‘F codes’ only (mental and behavioural disorders), but excluding non-specific diagnoses; for example, Z*, F99*, FXX.

e. Calculated based on the first accepted referral date to SLaM within the study window and the discharge date related to the latest accepted referral to SLaM within the study window.

f. Irrespective of whether patients received the standard rate or enhanced rate of the award.

Analyses adjusted for age, gender, ethnicity, deprivation and primary psychiatric diagnosis indicated that women were more likely to have received PIP (irrespective of type of PIP) than men (adjusted odds ratio (AOR) 1.07, 95% CI 1.04–1.10) (Table 2). PIP receipt increased with age, with those aged 35 years and older more likely to have received PIP than their younger counterparts. Compared with patients from a White background, those from a Black background and mixed/multiple ethnic and racial group were slightly more likely to have received PIP. There was one exception, as patients from an Asian/Asian British background were less likely to have received PIP (AOR 0.78, 95% CI 0.71–0.85) compared with patients from a White background. With regards to deprivation, those in the least deprived areas were less likely to have received PIP than patients who lived in more deprived areas. Compared with patients who had not received a primary psychiatric diagnosis, patients who had a primary psychiatric diagnosis of ‘other psychiatric disorders’ (e.g. eating disorders, ‘other’ perinatal psychiatric disorders) (AOR 0.59, 95% CI 0.54–0.65) were less likely to have received PIP. In contrast, PIP receipt was significantly higher across all other diagnoses when compared with patients who had not received a psychiatric diagnosis, with the strongest association found among patients diagnosed with intellectual disabilities (AOR 4.99, 95% CI 4.49–5.54). Considering only patients who had received a primary psychiatric diagnosis, those with an SMI diagnosis had 1.48 (95% CI 1.42–1.53) times the odds of receiving PIP compared with those who had no SMI diagnosis.

**Table 2:** Overview of sociodemographic and diagnostic patient characteristics and personal independence payment receipt (irrespective of type) between 2013 and 2019 (*N* = 143 714, of whom *n* = 37 120 had received a personal independence payment)

Characteristics	Never received PIP between 2013 and 2019, *n* (%)	Received PIP between 2013 and 2019, *n* (%)	Odds ratio (95% CI)	*P*-value	AOR^a^ (95% CI)	*P*-value
Gender						
Female	51 370 (48.2)	18 107 (48.8)	1.02 (1.00 – 1.05)	0.051	1.07 (1.04–1.10)	<0.001
Male	55 224 (51.8)	19 013 (51.2)	1		1	
Age (years)^b^						
18–24	19 815 (18.6)	4866 (13.1)	1		1	
25–34	33 114 (31.1)	8262 (22.3)	1.02 (0.98– 1.06)	0.431	1.03 (0.98–1.07)	0.243
35–44	26 746 (25.1)	9487 (25.6)	1.44 (1.39–1.50)	<0.001	1.41 (1.35–1.47)	<0.001
45–54	21 192 (19.9)	11 139 (30.0)	2.14 (2.06–2.23)	<0.001	1.96 (1.88–2.05)	<0.001
55–66	5727 (5.4)	3366 (9.1)	2.39 (2.27–2.52)	<0.001	2.20 (2.07–2.33)	<0.001
Ethnicity						
White	46 782 (53.7)	18 471 (57.1)	1		1	
Black/African/Caribbean/Black British	14 169 (16.3)	6354 (19.6)	1.13 (1.10–1.17)	<0.001	1.09 (1.05–1.13)	<0.001
Asian/Asian British	2410 (2.8)	713 (2.2)	0.75 (0.69–0.82)	<0.001	0.78 (0.71–0.85)	<0.001
Mixed/multiple racial and ethnic groups	2141 (2.5)	900 (2.8)	1.06 (0.98–1.15)	0.123	1.15 (1.06–1.25)	0.001
Other racial and ethnic minority groups	131 (0.2)	61 (0.2)	1.18 (0.87–1.60)	0.288	1.25 (0.91–1.72)	0.163
Not stated	21 483 (24.7)	5885 (18.2)	0.69 (0.67–0.72)	<0.001	0.84 (0.81–0.88)	<0.001
Deprivation (IMD quintile)^c^						
First (most deprived)	30 391 (29.9)	12 805 (36.2)	1		1	
Second	35 776 (35.2)	12 243 (34.6)	0.81 (0.79–0.84)	<0.001	0.83 (0.81–0.86)	<0.001
Third	19 621 (19.3)	5944 (16.8)	0.72 (0.69–0.75)	<0.001	0.74 (0.71–0.77)	<0.001
Fourth	9689 (9.5)	2690 (7.6)	0.66 (0.63–0.69)	<0.001	0.67 (0.64–0.71)	<0.001
Fifth (least deprived)	6163 (6.1)	1669 (4.7)	0.64 (0.61–0.68)	<0.001	0.66 (0.62–0.70)	<0.001
Primary psychiatric diagnosis categories (ICD-10 codes)^d^						
No primary psychiatric diagnosis recorded	37 453 (35.9)	9238 (25.8)	1		1	
Schizophrenia, schizotypal and delusional disorders (F20–29)	6073 (5.8)	4608 (12.9)	3.08 (2.94–3.22)	<0.001	2.83 (2.68–2.98)	<0.001
Severe mood disorders (i.e. bipolar affective disorder, severe or moderate depressive disorders, puerperal psychosis and postnatal depression (F30–31, F32.1–32.3, F33.1–33.3, F34.0–34.1, F53.0–53.1)	2637 (2.5)	1286 (3.6)	1.98 (1.84–2.12)	<0.001	1.82 (1.68–1.98)	<0.001
Anxiety, somatoform and stress-related disorders (F40–48)	13 813 (13.2)	5025 (14.0)	1.47 (1.42–1.53)	<0.001	1.40 (1.34–1.47)	<0.001
Other depressive disorders (F32.0, F32.8–32.9, F33.0, F33.4–33.9, F34.8–34.9, F38–39)	15 199 (14.6)	5233 (14.6)	1.40 (1.34–1.45)	<0.001	1.23 (1.17–1.28)	<0.001
Drug and alcohol-related disorders (F10–19, excluding F17)	17 034 (16.3)	5524 (15.4)	1.31 (1.27–1.37)	<0.001	1.20 (1.14–1.25)	<0.001
Personality disorders (F60–63)	1718 (1.7)	1263 (3.5)	2.98 (2.76–3.22)	<0.001	3.00 (2.75–3.28)	<0.001
Other psychiatric disorders (including eating disorders, other perinatal psychiatric disorders and ‘unspecified mental illness’) (F50–3, F53.8–53.9, F99)	4990 (4.8)	677 (1.9)	0.55 (0.51–0.60)	<0.001	0.59 (0.54–0.65)	<0.001
Intellectual disabilities (F70–79)	733 (0.7)	1018 (2.8)	5.63 (5.11–6.21)	<0.001	4.99 (4.49–5.54)	<0.001
Disorders of psychological development and behavioural and emotional disorders with onset usually occurring in childhood or adolescence (F80–89, F90–98)	4768 (4.6)	1925 (5.4)	1.64 (1.55–1.73)	<0.001	2.10 (1.98–2.24)	<0.001
Severe mental illness diagnosis (if yes to primary psychiatric diagnosis)						
No severe mental illness caseness	52 845 (78.4)	18 558 (69.4)	1		1	
Severe mental illness caseness (F2* (schizophrenia spectrum disorder), F30*/F31* (bipolar affective disorder) and F3* (affective disorder)	14 575 (21.6)	8197 (30.6)	1.60 (1.55–1.65)	<0.001	1.48 (1.42–1.53)	<0.001

Odds ratios, adjusted odds ratios and their corresponding 95% confidence intervals represent increase in odds of PIP receipt. PIP, personal independence payment; AOR, adjusted odds ratio; IMD, Index of Multiple Deprivation.

a. Adjusted for age (continuous), gender, ethnicity, deprivation and primary psychiatric diagnosis (yes/no).

b. Calculated at the PIP window start date (January 2013).

c. IMD scores published in 2015, patient postcode used closest before or after the PIP window start date (January 2013).

d. Earliest available within study window (January 2007 to December 2019), based on ICD-10 ‘F codes’ only (mental and behavioural disorders) but excluding non-specific diagnoses; for example, Z*, F99*, FXX.

A planned sensitivity analysis was conducted, exploring the impact of restricting the sample to patients who had resided in the SLaM catchment area (*n* = 95 660, of whom *n* = 23 259 received PIP at some point). The direction and strength of the associations found between the sociodemographic and diagnostic characteristics and PIP receipt were similar (Supplementary Table 1 available at https://doi.org/10.1192/bjo.2024.68).

Table 3 provides a descriptive overview of the sociodemographic and diagnostic profile of patients by type of PIP. Older patients (≥45 years of age) were overrepresented in the mobility only and both daily living and mobility categories compared with younger patients. A higher proportion of patients from a White background had received PIP mobility, when comparing this with the daily living and both daily living and mobility categories.

**Table 3:** Sociodemographic and diagnostic characteristics of patients by type of personal independence payment (daily living, mobility or both), data covering 2013–2019

Characteristics	Daily living only (*n* = 12 912), *n* (%)	Mobility only (*n* = 730), *n* (%)	Both daily living and mobility (*n* = 23 478), *n* (%)
Gender			
Female	5896 (45.7)	353 (48.4)	11 858 (50.5)
Male	7016 (54.3)	377 (51.6)	11 620 (49.5)
Age (years)^a^			
18–24	1931 (15.0)	99 (13.6)	2836 (12.1)
25–34	3506 (27.2)	173 (23.7)	4583 (19.5)
35–44	3398 (26.3)	188 (25.8)	5901 (25.1)
45–54	3317 (25.7)	211 (28.9)	7611 (32.4)
55–66	760 (5.9)	59 (8.1)	2547 (10.9)
Ethnicity			
White	6152 (54.9)	423 (66.1)	11 896 (57.9)
Black/African/Caribbean/Black British	2490 (22.2)	88 (13.8)	3767 (18.4)
Asian/Asian British	237 (2.1)	10 (1.6)	466 (2.3)
Mixed/multiple/other racial and ethnic groups	387 (3.5)	13 (2.0)	561 (2.7)
Not stated	1939 (17.3)	106 (16.6)	3840 (18.7)
Deprivation (IMD quintile)^b^			
First (most deprived)	4501 (36.8)	240 (34.2)	8064 (36.0)
Second	4342 (35.5)	248 (35.3)	7653 (34.1)
Third	2014 (16.5)	108 (15.4)	3822 (17.0)
Fourth	843 (6.9)	60 (8.6)	1787 (8.0)
Fifth (least deprived)	525 (4.3)	46 (6.6)	1098 (4.9)
Primary psychiatric diagnosis categories^c^ (ICD-10 codes)			
No primary psychiatric diagnosis recorded	2908 (22.9)	225 (31.8)	6105 (27.3)
Schizophrenia, schizotypal and delusional disorders (F20–29)	2222 (17.5)	43 (6.1)	2343 (10.5)
Severe mood disorders (i.e. bipolar affective disorder, severe or moderate depressive disorders, puerperal psychosis and postnatal depression (F30–31, F32.1–32.3, F33.1–33.3, F34.0–34.1, F53.0–53.1)	618 (4.9)	12 (1.7)	656 (2.9)
Anxiety, somatoform and stress-related disorders (F40–48)	1456 (11.5)	135 (19.1)	3434 (15.3)
Other depressive disorders (F32.0, F32.8–32.9, F33.0, F33.4–33.9, F34.8–34.9, F38–39).	1733 (13.7)	86 (12.2)	3414 (15.3)
Drug and alcohol-related disorders (F10–19, excluding F17)	2195 (17.3)	143 (20.2)	3186 (14.2)
Personality disorders (F60–63)	512 (4.0)	14 (2.0)	737 (3.3)
Other psychiatric disorders (including eating disorders, other perinatal psychiatric disorders and ‘unspecified mental illness’) (F50–3, F53.8–53.9, F99)	287 (2.3)	16 (2.3)	374 (1.7)
Intellectual disabilities (F70–79)	45 (0.4)	<5 (0.0)	970 (4.3)
Disorders of psychological development and behavioural and emotional disorders with onset usually occurring in childhood or adolescence (F80–89, F90–98)	720 (5.7)	31 (4.4)	1174 (5.2)
Severe mental illness diagnosis (if yes to primary psychiatric diagnosis)			
No severe mental illness diagnosis	6228 (63.1)	406 (82.9)	11 924 (72.7)
Severe mental illness diagnosis (F2* (schizophrenia-spectrum disorder), F30*/F31* (bipolar affective disorder) and F3* (affective disorder)	3638 (36.9)	84 (17.1)	4475 (27.3)

IMD: Index of Multiple Deprivation; PIP, personal independence payment.

a. Calculated at the PIP window start date (January 2013).

b. IMD scores published in 2015, patient postcode used closest before or after the PIP window start date (January 2013).

c. Earliest available within study window (January 2007 to December 2019), based on ICD-10 ‘F codes’ only (mental and behavioural disorders) but excluding non-specific diagnoses; for example, Z*, F99*, FXX.

Adjusted analyses indicated that women were more likely to have received the PIP daily living only or both the daily living and mobility PIP than men (Supplementary Table 2). Compared with patients from a White background, patients from Black or minority ethnic background had lower odds of having received PIP mobility only. An SMI diagnosis was associated with a higher odds of PIP daily living only as well as both daily living and mobility PIP, but a negative association was found with PIP mobility only.

## Discussion

This study explored how PIP receipt, including type of PIP, varied over a 7-year period among working-age individuals who accessed mental health services, and factors associated with PIP receipt. As expected, considering the phased roll out of PIP replacing DLA from 2013 onward, levels of PIP steadily increased over time, from *n* = 1671 (1.2%) in 2013 to *n* = 33 968 (23.6%) in 2019. On a national level, about 6% of the working-age population are receiving disability benefits, whether this is PIP or DLA.^15^ This indicates that people who access mental health services are approximately four times more likely to be in receipt of these kinds of benefits. It is important to note that our estimate did not include those who were still on DLA, so this difference is likely to be even greater (see Supplementary Fig. 1). In 2019, an additional 13 847 patients were still in receipt of DLA. Indeed, we know that psychiatric conditions are the most commonly recorded disabling conditions for PIP claims.^16^ A similar upward trend was seen when exploring the different types of PIP over the same period. Our findings indicate that nearly three in five patients had received both PIP daily living and mobility, followed by one in three who had received daily living only.

Our results indicated that PIP receipt was slightly elevated in women compared with men. In the working-age population men are more likely to be in receipt of disability benefits.^15^ We know from previous research that men are more likely to be diagnosed with substance misuse, whereas women are more likely to be diagnosed with eating disorders and depression. In addition, some research indicates that women have higher levels of mental health comorbidities than men, leading to more severe symptoms and disability.^17,18^ As such, women might be more likely to successfully claim PIP.

We found that people living in more deprived areas were more likely to receive PIP, despite the fact that PIP is not means tested. Plausible explanations could include a lack of awareness around PIP eligibility criteria. For example, there is a longstanding misunderstanding that PIP and DLA are out-of-work benefits, which is not the case, so people in work may mistakenly think they are ineligible.^19^ In contrast, it may also be caused by PIP take-up being partial, like for other benefits. People may believe they are eligible, but do not apply because of stigma or the perceived burden of claiming. People in deprived areas may be more likely to claim, not because they stigmatise benefits less, but because their financial needs are greater.^20,21^ Nevertheless, our findings may also be partly explained by residual confounding (e.g. by education or work status), as we know that there is a strong social gradient in relation to long-term health conditions and disability.^22^ It could also be that SMIs are concentrated in more deprived areas.

To the best of our knowledge, this is the first time that detailed large-scale data on ethnicity have been made available in relation to PIP receipt. A mixed picture emerged, as PIP receipt was lower in patients from an Asian background when compared with patients from a White background, whereas Black patients and those from a mixed/multiple ethnic and racial group were more likely to have received PIP, albeit this association was weak (Black: AOR 1.09, 95% CI 1.05–1.13; mixed/multiple ethnic group: AOR 1.15, 95% CI 1.06–1.25). It is well documented that certain ethnic and racial minority groups face structural inequalities that may increase their vulnerability toward developing long-term health conditions, including mental disorders, and subsequent disabilities.^23,24^ It would be interesting to explore whether benefits-related stigma differs among people from different racial and ethnic minority groups. It is important to acknowledge that we had missing data for ethnicity, as this is often poorly recorded in electronic health records. This needs to be taken into consideration when interpreting the findings.

Across the board, PIP receipt was higher among patients with a diagnosed mental disorder compared with those who had accessed SLaM services but had not received a diagnosis. The exception was the category ‘other psychiatric disorders’, including eating disorders and ‘other’ perinatal disorders, as patients had 0.59 odds (95% CI 0.54–0.65) of PIP receipt. Considering the nature of perinatal disorders, they are most likely to be of a shorter duration, whereas this is not the case for eating disorders. Future research is needed on why PIP uptake is lower among this patient group. The strength of the association was strongest for intellectual disabilities (AOR 4.99, 95% CI 4.49–5.54) and personality disorders (AOR 3.00, 95% CI 2.75–3.28), which could possibly be explained by the chronicity of these disorders as well as their severe impact on an individual's functional abilities. This explanation can also be extended to the moderate association seen between SMI and PIP receipt. It is important to acknowledge that a medical diagnosis is not required to be judged eligible for PIP, as the PIP assessment focuses on a person's functional ability. As such, although we used a psychiatric diagnosis as a proxy for severity, future research could benefit from exploring other indicators of functional impairment, including in-patient admissions and emergency care presentations that include a psychiatric evaluation, and explore whether the observed associations remain consistent.

### Implications and future research

The UK Government is planning to use the PIP assessment to replace the WCA that currently forms part of the claims process for Universal Credit.^9^ It is important that the PIP assessment is appropriate for those in need of support, irrespective of the particulars of their health condition or disability. One of the few available quantitative studies found that when more WCA re-assessments had taken place on a local authority level, a higher likelihood of suicides, self-reported mental health problems and antidepressant prescriptions was found.^25^ So far, however, all that can be concluded from this study is that there are temporal links, for example, between disability assessments and worsening mental health, but such time trends are susceptible to the ecological fallacy. Without individual-level data it is impossible to decide whether these proposed links are coincidental or causal.

Our novel, individual-level, linked longitudinal data-set does hold some information on PIP re-assessments as well as mental health service utilisation. Hence, our future research will focus on exploring the patterns and frequency of these re-assessments among people who access mental health services. In addition, we have information on whether patients were in receipt of DLA before they received PIP. Although we will not know whether their application for PIP was rejected, it is possible for us to examine the effects of migration from DLA to PIP among those who did transition successfully. It is also essential to explore whether the migration from DLA to PIP has disproportionality affected more vulnerable patient groups, such as those from a racial and ethnic minority background, older patients, those with lower educational attainment and patients with fluctuating mental disorders, as they may face a cumulative disadvantage navigating the claims process.^26^

Another change that warrants further research is the duration of the awards given since the implementation of PIP. Approximately four in five new PIP claimants received an award of 2 years or less, whereas those re-assessed as part of the migration from DLA to PIP appear to receive awards for a longer duration.^16^ Furthermore, the number of ongoing awards, meaning awards that technically have no end date and only a light touch review is planned at the 10-year point, has reduced drastically under PIP. About one in five new PIP claims were ongoing, compared with about two in five among former DLA recipients. Bearing in mind the chronicity and severity of certain mental disorders, in particular SMI, further investigations are needed to explore whether the imposed mandatory PIP re-assessments, irrespective of whether an individual's circumstances have changed, and the trend to award PIP for a shorter duration are justified. It is key to keep in mind not only the costs related to these re-assessments for the public purse, but also the considerable emotional toll it takes on claimants,^27^ especially when it is unlikely that for certain patient groups, their functional ability has changed. Using our data-set, we will be able to explore the average duration of a PIP award by psychiatric diagnosis. Consequently, our findings could be used to inform whether there are certain psychiatric diagnoses for which the frequency of the re-assessments could be eased, or alternatively, whether some conditions could be recommended for a light touch review only. The UK Government does have a strategy whereby severe and progressive conditions that are unlikely to get better may trigger a light touch review only. However, how well this strategy is tailored to mental disorders is currently unclear.^9,28^ Unfortunately, we do not hold information on the intended duration of the PIP award, but only the actual duration of PIP receipt.

### Strengths and limitations

The main strength lies in the use of a unique, large-scale, individual-level linked data-set that includes detailed information on the mental health treatment pathways of patients and their interactions with the UK welfare system. A limitation is that mental health record data were derived from a single mental health service provider. The foundational profile of PIP receipt we describe may not, therefore, generalise to more rural areas or areas with a lower ethnic density. It is well documented that disability benefit receipt is concentrated in certain geographical areas in the UK.^22^ Another limitation is that the information we had access to regarding PIP receipt was at claim receipt level, meaning that it did not show any in-claim activity; for example, if patients lost the enhanced rate of their PIP mobility component during a PIP award. However, consultations with our advisory group of people with lived experience indicated that changes to the type of award are rare without a re-assessment.

In conclusion, we found that over a 7-year period, one in four people who access mental health services had received PIP at some point, and most had received both the PIP daily living and mobility component. Our findings indicated that patients who are likely to be most in need, as indicated by their psychiatric diagnosis, had a high likelihood of PIP receipt. The linked data-set underpinning this paper is an important asset in further solidifying the evidence base concerning disability benefit receipt among people who experience mental health problems, trends over time and the impact of welfare reform on a whole range of health, work and treatment outcomes.

## Supporting information

Stevelink et al. supplementary material Stevelink et al. supplementary material

## Data Availability

Data are not publicly available. Access to deidentified data can be applied for via the NIHR Maudsley Biomedical Research Centre at the South London and Maudsley NHS Foundation Trust, upon reasonable request. Requests for data will be considered on a case-by-case basis, given the sensitive nature of the data, and access will only be granted if approval is given by the Work and Health Screening Panel and other governance requirements are fulfilled. For more information, please contact: cris.administrator@slam.nhs.uk.
