# Supplementary material for: Personal independence payments among people who access mental health services: results from a novel data linkage
Source: BJPsych Open. 2024 Sep 24;10(5):e150. doi: 10.1192/bjo.2024.68 (PMC11457205; doi:10.1192/bjo.2024.68)

*Supplementary Table 1: Overview of characteristics of study population* ***who lived in SLAM catchment area*** *by PIP receipt within the PIP time window (2013-2019) (n=95660 of whom n=23259 received PIP at some point). Odds ratio (OR) , Adjusted odds ratios (AOR) and their corresponding 95% intervals (CI) represent increase in odds of PIP receipt.*

| **Characteristics** | **Never received PIP between 2013-2019**  **N (%)** | **Received PIP between 2013-2019**  **N (%)** | **OR (95% CI)** | **p-value** | **AOR# (95% CI)** | **p-value** |
| --- | --- | --- | --- | --- | --- | --- |
| **Sex** |  |  |  |  |  |  |
| Female | 37183 (51.4) | 11730 (50.4) | 0.96 (0.94 – 0.99) | 0.014 | 1.05 (1.02 – 1.09) | 0.003 |
| Male | 35218 (48.6) | 11529 (49.6) | 1 |  | 1 |  |
| **Age (years)**^ |  |  |  |  |  |  |
| 18-24 | 12622 (17.4) | 2509 (10.8) | 1 |  | 1 |  |
| 25-34 | 22554 (31.2) | 4791  (20.6) | 1.07 (1.01 – 1.13) | 0.001 | 1.11 (1.05 – 1.18) | 0.002 |
| 35-44 | 18426 (25.5) | 5860 (25.2) | 1.60 (1.52 – 1.69) | <0.001 | 1.61 (1.52 – 1.71) | <0.001 |
| 45-54 | 14781 (20.4) | 7640 (32.9) | 2.60 (2.47 – 2.74) | <0.001 | 2.44 (2.31 – 2.59) | <0.001 |
| 55-66 | 4018 (5.6) | 2459 (10.6) | 3.08 (2.88 – 3.29) | <0.001 | 2.97 (2.76 – 3.19) | <0.001 |
| **Ethnicity** |  |  |  |  |  |  |
| White | 29633 (52.0) | 10569 (53.8) | 1 |  | 1 |  |
| Black/African/Caribbean/  Black British | 12180 (21.4) | 5566 (28.4) | 1.28 (1.23 – 1.33) | <0.001 | 1.26 (1.21 – 1.31) | <0.001 |
| Asian/Asian British | 1720 (3.0) | 513 (2.6) | 0.84 (0.76 – 0.93) | 0.001 | 0.89 (0.80 – 0.99) | 0.027 |
| Mixed/Multiple  racial and ethnic groups | 1681 (3.0) | 715 (3.6) | 1.19 (1.09 – 1.31) | <0.001 | 1.37 (1.25 – 1.51) | <0.001 |
| Other racial and ethnic minority groups | 99 (0.2) | 39 (0.2) | 1.10 (0.76 – 1.60) | 0.600 | 1.17 (0.80 – 1.71) | 0.424 |
| Not stated | 11689 (20.5) | 2230 (11.4) | 0.53 (0.51 – 0.56) | <0.001 | 0.70 (0.66 – 0.73) | <0.001 |
| **Deprivation (IMD quintile)**¥ |  |  |  |  |  |  |
| First (most deprived) | 25203 (35.2) | 9718 (42.5) | 1 |  | 1 |  |
| Second | 27614 (38.6) | 8620 (37.7) | 0.81 (0.78 – 0.84) | <0.001 | 0.84 (0.80 – 0.87) | <0.001 |
| Third | 13066 (18.3) | 3340 (14.6) | 0.66 (0.63 – 0.69) | <0.001 | 0.69 (0.65 – 0.72) | <0.001 |
| Fourth | 3877 (5.4) | 824 (3.6) | 0.55 (0.51 – 0.60) | <0.001 | 0.56 (0.51 – 0.61) | <0.001 |
| Fifth (least deprived) | 1778 (2.5) | 379 (1.7) | 0.55 (0.49 – 0.62) | <0.001 | 0.58 (0.51 – 0.65) | <0.001 |
| **Primary psychiatric diagnosis**  **Diagnosis categories (ICD-10 codes)**$ |  |  |  |  |  |  |
| No primary psychiatric diagnosis recorded | 23727 (33.4) | 4433 (19.8) | 1 |  | 1 |  |
| Schizophrenia, schizotypal and delusional disorders (F20-F29) | 4924 (6.9) | 3572 (15.9) | 3.88 (3.68 – 4.10) | <0.001 | 3.29 (3.09 – 3.51) | <0.001 |
| Severe mood disorders (i.e. bipolar affective disorder, severe or moderate depressive disorders, puerperal psychosis and postnatal depression (F30-31, F32.1-32.3, F33.1-33.3, F34.0-34.1, F53.0-53.1) | 2106 (3.0) | 924 (4.1) | 2.35 (2.16 – 2.55) | <0.001 | 2.11 (1.91 – 2.32) | <0.001 |
| Anxiety, somatoform and stress-related disorders (F40-48) | 9797 (13.8) | 3027 (13.5) | 1.65 (1.57 – 1.74) | <0.001 | 1.55 (1.46 – 1.64) | <0.001 |
| Other depressive disorders (F32.0, F32.8-32.9, F33.0, F33.4-33.9, F34.8-34.9, F38-39). | 13038 (18.4) | 4408 (19.7) | 1.81 (1.73 – 1.90) | <0.001 | 1.60 (1.52 – 1.69) | <0.001 |
| Drug and alcohol-related disorders (F10-19, excluding F17) | 10291 (14.5) | 3413 (15.2) | 1.78 (1.69 – 1.87) | <0.001 | 1.56 (1.47 – 1.65) | <0.001 |
| Personality disorders (F60-63) | 1355 (1.9) | 829 (3.7) | 3.27 (2.99 – 3.59) | <0.001 | 3.40 (3.06 – 3.78) | <0.001 |
| Other psychiatric disorders (including eating disorders, other perinatal psychiatric disorders, and ‘unspecified mental illness’) (F50-3, F53.8-53.9, F99) | 3253 (4.6) | 326 (1.5) | 0.54 (0.48 – 0.60) | <0.001 | 0.59 (0.51 – 0.67) | <0.001 |
| Intellectual disabilities (F70-F79) | 649 (0.9) | 928 (4.1) | 7.65 (6.89 – 8.50) | <0.001 | 6.86 (6.11 – 7.69) | <0.001 |
| Disorders of psychological development and behavioural and emotional disorders with onset usually occurring in childhood or adolescence (F80-89, F90-98) | 1818 (2.6) | 562 (2.5) | 1.65 (1.50 – 1.83) | <0.001 | 2.40 (2.15 – 2.68) | <0.001 |
| **If yes to primary psychiatric diagnosis**  **Diagnosis categories (ICD-10 codes)** |  |  |  |  |  |  |
| No severe mental illness caseness | 35556 (74.8) | 11730 (64.8) | 1 |  | 1 |  |
| Severe mental illness caseness (F2* (schizophrenia-spectrum disorder), F30*/F31* (bipolar affective disorder) and F3* (affective disorder) | 11.953 (25.2) | 6373 (35.2) | 1.62 (1.56 – 1.68) | <0.001 | 1.47 (1.40 – 1.53) | <0.001 |

IMD: Index of Multiple Deprivation. PIP: Personal Independence Payment. ^ Calculated at the PIP window start date (January 2013). ¥ IMD scores published in 2015, patient postcode used closest before or after the PIP window start date (January 2013). $ Earliest available within study window (January 2007 to December 2019), based on ICD-10 ‘F codes’ only (mental and behavioural disorders) but excluding non-specific diagnoses, for example, Z*, F99*, FXX.

*Supplementary Table 2: Unadjusted and adjusted multinominal regression results regarding PIP receipt by type of award (daily living only, mobility only or both) within the PIP time window (2013-2019) (n=37120). Reference category: never received PIP between 2013 and 2019. Odds ratio (OR) , Adjusted odds ratios (AOR) and their corresponding 95% intervals (CI) represent increase in odds of PIP receipt.*

|  | **Unadjusted** | | | **Adjusted** | | |
| --- | --- | --- | --- | --- | --- | --- |
| **Characteristics** | **Received PIP daily living award between 2013-2019** | **Received PIP - mobility award between 2013-2019** | **Received both PIP daily living award and PIP mobility between 2013-2019** | **Received PIP daily living award between 2013-2019** | **Received PIP - mobility award between 2013-2019** | **Received both PIP daily living award and PIP mobility between 2013-2019** |
|  | OR (95% CI), p value | | | AOR# (95% CI), p value | | |
| **Sex** |  |  |  |  |  |  |
| Female | 0.90 (0.87 - 0.94), <0.001 | 1.01 (0.87 - 1.16), 0.930 | 1.10 (1.07 - 1.13), <0.001 | 0.92 (0.88 - 0.96), <0.001 | 1.05 (0.90 - 1.23), 0.548 | 1.16 (1.12 - 1.19), <0.001 |
| Male | 1 | 1 | 1 | 1 | 1 | 1 |
| **Age (years)**^ |  |  |  |  |  |  |
| 18-24 | 1 | 1 | 1 | 1 | 1 | 1 |
| 25-34 | 1.09 (1.02 - 1.15), 0.005 | 1.05 (0.82 - 1.34), 0.724 | 0.97 (0.92 - 1.02), 0.189 | 1.08 (1.02 - 1.16), 0.013 | 1.14 (0.87 – 1.50), 0.351 | 0.98 (0.93 - 1.04), 0.520 |
| 35-44 | 1.30 (1.23 - 1.38), <0.001 | 1.41 (1.10 - 1.80), 0.006 | 1.54 (1.47 - 1.62), <0.001 | 1.23 (1.15 - 1.31), <0.001 | 1.63 (1.25 – 2.13), <0.001 | 1.52 (1.44 - 1.60), <0.001 |
| 45-54 | 1.61 (1.51 - 1.70), <0.001 | 1.99 (1.57 - 2.53), <0.001 | 2.51 (2.39 - 2.63), <0.001 | 1.43 (1.34 – 1.53), <0.001 | 2.00 (1.53 - 2.61), <0.001 | 2.32 (2.20 – 2.44), <0.001 |
| 55-66 | 1.36 (1.25 - 1.49), 0.108 | 2.06 (1.49 - 2.85), 0.003 | 3.11 (2.92 - 3.30), <0.001 | 1.22 (1.10 – 1.34), 0.001 | 2.06 (1.44 - 2.93), <0.001 | 2.87 (2.68 – 3.07), <0.001 |
| **Ethnicity**~ |  |  |  |  |  |  |
| White | 1 | 1 | 1 | 1 | 1 | 1 |
| Black/African/Caribbean/  Black British | 1.34 (1.27 - 1.41), <0.001 | 0.69 (0.55 - 0.86), 0.001 | 1.05 (1.00 - 1.09), 0.034 | 1.27 (1.20 - 1.34), <0.001 | 0.66 (0.52 - 0.84), <0.001 | 1.01 (0.97 - 1.06), 0.537 |
| Asian/Asian British | 0.75 (0.65 - 0.86), 0.001 | 0.46 (0.24 - 0.86), 0.015 | 0.76 (0.69 - 0.84), <0.001 | 0.76 (0.66 - 0.87), <0.001 | 0.48 (0.26 - 0.90), 0.022 | 0.80 (0.72 - 0.89), <0.001 |
| Mixed/Multiple/other  racial and ethnic minority groups | 1.30 (1.16 - 1.45), <0.001 | 0.63 (0.36 - 1.10), 0.105 | 0.97 (0.88 - 1.07), 0.542 | 1.30 (1.16 - 1.46), <0.001 | 0.70 (0.40 - 1.22), 0.208 | 1.09 (0.99 - 1.20), 0.078 |
| Not stated | 0.69 (0.65 - 0.72), <0.001 | 0.55 (0.44 - 0.68), <0.001 | 0.70 (0.68 - 0.73), <0.001 | 0.84 (0.80 - 0.89), <0.001 | 0.58 (0.46 - 0.73), <0.001 | 0.85 (0.82 - 0.89), <0.001 |
| **Deprivation (IMD quintile)**¥ |  |  |  |  |  |  |
| First (most deprived) | 1 | 1 | 1 | 1 | 1 | 1 |
| Second | 0.82 (0.78 - 0.86), <0.001 | 0.88 (0.73 - 1.05), 0.152 | 0.81 (0.78 - 0.83), <0.001 | 0.85 (0.81 - 0.89), <0.001 | 0.87 (0.72 - 1.05), 0.115 | 0.82 (0.79 - 0.85), <0.001 |
| Third | 0.69 (0.66 - 0.73), <0.001 | 0.70 (0.56 - 0.88), 0.002 | 0.73 (0.70 - 0.77), <0.001 | 0.73 (0.69 - 0.78), <0.001 | 0.68 (0.53 - 0.86), 0.002 | 0.75 (0.72 - 0.78), <0.001 |
| Fourth | 0.59 (0.54 - 0.63), <0.001 | 0.78 (0.59 - 1.04), 0.093 | 0.70 (0.66 - 0.74), <0.001 | 0.62 (0.57 - 0.68), <0.001 | 0.70 (0.52 – 0.95), 0.022 | 0.70 (0.66 - 0.74), <0.001 |
| Fifth (least deprived) | 0.58 (0.52 - 0.63), <0.001 | 0.95 (0.69 - 1.30), 0.727 | 0.67 (0.63 - 0.72), <0.001 | 0.62 (0.56 - 0.68), <0.001 | 0.83 (0.59 - 1.16), 0.278 | 0.68 (0.63 - 0.73), <0.001 |
| **Primary psychiatric diagnosis**  **Diagnosis categories (ICD-10 codes)**$ |  |  |  |  |  |  |
| No primary psychiatric diagnosis recorded | 1 | 1 | 1 | 1 | 1 | 1 |
| Schizophrenia, schizotypal and delusional disorders (F20-F29) | 4.71 (4.43 – 5.01), <0.001 | 1.18 (0.85 - 1.64), 0.325 | 2.37 (2.24 – 2.50), <0.001 | 4.45 (4.14 - 4.79), <0.001 | 1.02 (0.69 - 1.50), 0.922 | 2.12 (1.99 – 2.27), <0.001 |
| Severe mood disorders (i.e. bipolar affective disorder, severe or moderate depressive disorders, puerperal psychosis and postnatal depression (F30-31, F32.1-32.3, F33.1-33.3, F34.0-34.1, F53.0-53.1) | 3.02 (2.74 - 3.32), <0.001 | 0.76 (0.42 - 1.36), 0.350 | 1.53 (1.40 - 1.67), <0.001 | 3.00 (2.69 - 3.34), <0.001 | 0.52 (0.26 – 1.06), 0.072 | 1.36 (1.23 - 1.50), 0.002 |
| Anxiety, somatoform and stress-related disorders (F40-48) | 1.36 (1.27 - 1.45), <0.001 | 1.63 (1.31 – 2.02), <0.001 | 1.53 (1.46 - 1.60), <0.001 | 1.33 (1.23 - 1.43), <0.001 | 1.49 (1.17 – 1.89), 0.001 | 1.43 (1.36 - 1.51), <0.001 |
| Other depressive disorders (F32.0, F32.8-32.9, F33.0, F33.4-33.9, F34.8-34.9, F38-39). | 1.47 (1.38 - 1.56), <0.001 | 0.94 (0.73 - 1.21), 0.638 | 1.38 (1.32 - 1.44), <0.001 | 1.36 (1.27 - 1.46), <0.001 | 0.79 (0.60 - 1.05), 0.104 | 1.18 (1.12 - 1.25), <0.001 |
| Drug and alcohol-related disorders (F10-19, excluding F17) | 1.66 (1.57 - 1.76), <0.001 | 1.40 (1.13 - 1.72), 0.002 | 1.15 (1.10 - 1.20), <0.001 | 1.60 (1.49 - 1.71), <0.001 | 1.12 (0.88 - 1.42), 0.357 | 1.02 (0.97 - 1.08), 0.416 |
| Personality disorders (F60-63) | 3.84 (3.45 – 4.27), <0.001 | 1.36 (0.79 – 2.33), 0.270 | 2.63 (2.40 - 2.88), <0.001 | 3.88 (3.44 – 4.37), <0.001 | 1.25 (0.70 – 2.26), 0.452 | 2.66 (2.40 - 2.95), <0.001 |
| Other psychiatric disorders (including eating disorders, other perinatal psychiatric disorders, and ‘unspecified mental illness’) (F50-3, F53.8-53.9, F99) | 0.74 (0.65 - 0.84), <0.001 | 0.53 (0.32 - 0.89), 0.015 | 0.46 (0.41 - 0.51), <0.001 | 0.81 (0.70 – 0.92), 0.002 | 0.55 (0.32 – 0.93), 0.026 | 0.50 (0.44 - 0.56), <0.001 |
| Intellectual disabilities (F70-F79) | 0.79 (0.58 – 1.07), 0.129 | 0.68 (0.22 – 2.13), 0.510 | 8.12 (7.35 – 8.97), <0.001 | 0.72 (0.52 - 0.99), 0.43 | 0.65 (0.21 – 2.04), 0.462 | 7.28 (6.53 – 8.13), <0.001 |
| Disorders of psychological development and behavioural and emotional disorders with onset usually occurring in childhood or adolescence (F80-89, F90-98) | 1.94 (1.78 – 2.12), <0.001 | 1.08 (0.74 - 1.58), 0.681 | 1.51 (1.41 - 1.62), <0.001 | 2.27 (2.07 - 2.49), <0.001 | 1.38 (0.94 – 2.04), 0.101 | 2.05 (1.90 – 2.21), <0.001 |
| **If yes to primary psychiatric diagnosis**  **diagnosis categories (ICD-10 codes)** |  |  |  |  |  |  |
| No severe mental illness caseness | 1 | 1 | 1 | 1 | 1 | 1 |
| Severe mental illness caseness (F2* (schizophrenia-spectrum disorder), F30*/F31* (bipolar affective disorder) and F3* (affective disorder) | 2.12 (2.03 – 2.22), <0.001 | 0.75 (0.59 – 0.95), 0.017 | 1.36 (1.31 - 1.41), <0.001 | 2.01 (1.91 - 2.11), <0.001 | 0.68 (0.52 – 0.90), 0.006 | 1.23 (1.17 - 1.29), <0.001 |

AOR: Adjusted Odds Ratio. IMD: Index of Multiple Deprivation. OR: Odds ratio. PIP: Personal Independence Payment. ^ Calculated at the PIP window start date (January 2013). ¥ IMD scores published in 2015, patient postcode used closest before or after the PIP window start date (January 2013). ~ Due to small numbers, mixed/multiple racial and ethnic groups category was combined with the other racial and ethnic minority groups category. $ Earliest available within study window (January 2007 to December 2019), based on ICD-10 ‘F codes’ only (mental and behavioural disorders) but excluding non-specific diagnoses, for example, Z*, F99*, FXX. # Adjusted for age (continuous), sex, ethnicity, deprivation and primary psychiatric diagnosis (yes/no).

*Supplementary Graph 1: Number of patients who received PIP (irrespective of type of PIP) or DLA (irrespective of type of DLA) by calendar year (N=143714), data covering 2013-2019.*


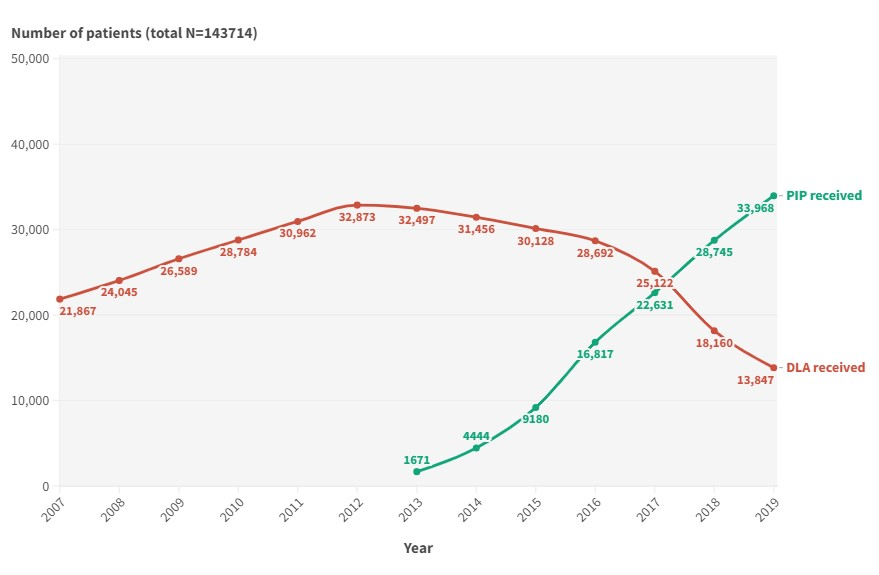

Supplement: Stevelink et al. supplementary material [file S2056472424000681sup001.docx]
